# Probing the Double-Layered Cotyledon Cell Structure of Navy Beans: Barrier Effect of the Protein Matrix on In Vitro Starch Digestion

**DOI:** 10.3390/nu15010105

**Published:** 2022-12-26

**Authors:** Duc Toan Do, Jaspreet Singh, Stuart Johnson, Harjinder Singh

**Affiliations:** 1 Riddet Institute, Massey University, Private Bag 11 222, Palmerston North 4442, New Zealand; 2 School of Food and Advanced Technology, Massey University, Private Bag 11 222, Palmerston North 4442, New Zealand; 3 School of Molecular and Life Sciences, Faculty of Science and Engineering, Curtin University, Bentley 6102, Australia

**Keywords:** cotyledon cells, in vitro starch digestion, legume structure, low glycaemic index, navy beans, protein matrix

## Abstract

The microstructure of legumes plays a crucial role in regulating starch digestion and postprandial glycemic responses. Starch granules are double encapsulated within the outer cell wall and the inner protein matrix of legume cotyledon cells. Despite progress in understanding the role of cell walls in delaying starch digestion, the role of the protein matrix has received little research attention. The aim of this study was to evaluate if the protein matrix and cell wall may present combined physical barriers retarding enzyme hydrolysis of intracellular starch. Intact cotyledon cells were isolated from navy beans and used to assess the barrier effect of the protein matrix on the digestion of starch under conditions simulating the upper gastrointestinal tract. The cells were pretreated with pepsin at 37 °C and pH 2.0 for 1, 4, or 24 h and without pepsin for 24 h (control) to facilitate removal of the intracellular protein matrix prior to cooking and simulated in vitro digestion. A longer pretreatment time resulted in a lower protein content of the cells and a higher initial rate and extent of starch hydrolysis. We suggest that in addition to the primary cell wall barrier, the protein matrix provides a secondary barrier restricting the accessibility of α-amylase to starch. This study provides a new fundamental understanding of the relationship between the structural organization of legume cotyledon cells and starch digestion that could inform the design of novel low glycemic index foods.

## 1. Introduction

Epidemiologic evidence supports the association of consumption of nutrient-dense, low glycaemic index legumes with a decreased risk of incident type 2 diabetes and improved glycaemic control in diabetic individuals [1,2,3]. Starchy legumes (e.g., beans, peas, chickpeas, lentils, etc.) contain a substantial proportion of slowly digestible starch and physically inaccessible resistant starch. These starch fractions are believed to slow the rate of carbohydrate release in vitro and, consequently, lower the postprandial blood glucose response in vivo [4,5,6].

An accumulating body of in vitro and in vivo evidence has firmly established a link between the microstructure of food legumes and the slow digestion properties of their starch [7,8,9,10,11,12]. The cotyledon tissue of starchy legumes comprises numerous cells within which starch granules are tightly embedded in protein matrices and all encapsulated by thick, strong cell walls [13]. In an earlier study, Berg et al. [7] showed that the entrapment of multiple starch granules within cotyledon cells reduced the in vitro hydrolysis rate and the extent of starch in cooked whole navy beans. This is because the resilient cell walls remained mostly intact throughout cooking and subsequent digestion and, thus, could act as physical barriers obstructing free access of α-amylase to the intracellular starch.

Recently, there has been a surge of interest in the use of intact, isolated cotyledon cells as a food model to gain a deeper mechanistic understanding of how microscale legume structure controls in vitro starch digestion [9,10,11,14]. Bhattarai, Dhital, Wu, Chen, and Gidley [15] studied the digestive behaviour of isolated legume cells using a dynamic in vitro rat stomach duodenum (DIVRSD) model. The authors proposed three possible mechanisms that may explain the limited hydrolysis of starch inside the cells. Firstly, cell wall intactness reduces the accessibility of digestive enzymes to starch during simulated digestion. Secondly, the swelling and gelatinisation of entrapped starch granules are restricted during cooking, resulting in the retention of the enzyme-resistant crystalline starch structure. Thirdly, the noncatalytic binding of α-amylase to cell wall components prevents its binding to the preferred starch substrate. New evidence further suggests that cell wall porosity controls the diffusion of digestive enzymes and the release of hydrolysed products, which can significantly affect the digestion of starch inside isolated cells from plant foods [16].

To date, most studies have identified the cell wall as a primary physical barrier to starch digestion [7,9,13,17]. Despite the fact that the cell wall coexists with the protein matrix within the cotyledon cell and both naturally encapsulate starch granules [13], the barrier effect exerted by the protein matrix on modulating starch digestion has received inadequate investigation. Rovalino-Córdova, Fogliano, and Capuano [18] and Rovalino-Córdova, Fogliano, and Capuano [19] revealed that the in vitro rate and/or extent of starch hydrolysis of intact red kidney bean cells was significantly reduced in the absence of one or more digestive proteases (i.e., pepsin, trypsin, and chymotrypsin) that were able to hydrolyse the protein matrix. This clearly indicates that the protein matrix represents an additional structural barrier to starch digestion [19]. It has been hypothesized that the compact protein network surrounding starch granules could impede α-amylase mobility within the cells and decrease the starch surface area available for amylase binding and catalysis [18].

The present study aims to extend our understanding of the role of the protein matrix in determining the kinetics of starch digestion using isolated navy bean cotyledon cells as a food model. Navy beans were selected as the legume variety for investigation due to previous evidence supporting the strong relationship between their cotyledon cell structures and starch digestion in vitro [7,11]. In this study, the cells were pretreated with pepsin at 37 °C, pH 2.0, and various incubation times to facilitate the removal of the protein barrier while retaining both the starch granules and the cell walls in an intact state. Accordingly, cell samples with different protein matrix microstructures were generated. Following the cooking of the pepsin-treated cells, the gastrointestinal (GI) fate of the encapsulated starch was assessed using a static in vitro digestion model. It was postulated that the enzymatic breakdown of the intracellular protein matrix could enhance starch hydrolysis efficiency.

## 2. Materials and Methods

### 2.1. Materials

One batch of raw navy beans (*Phaseolus vulgaris* L.) was procured from a local store in Palmerston North, New Zealand. Pepsin (from porcine gastric mucosa, ≥250 units/mg protein), pancreatin (from porcine pancreas, activity equivalent to 4× USP specifications), invertase (from baker’s yeast (*S. cerevisiae*), Grade VII, ≥300 units/mg solid), porcine pancreatic α-amylase (from porcine pancreas, type VI-B), soluble potato starch (S-2630), and maltose (S-5885) were purchased from Sigma-Aldrich Ltd., St Louis, MO, USA. Amyloglucosidase (for total dietary fibre and starch assays, 3260 U/mL) was purchased from Megazyme International Ireland Ltd., Wicklow, Ireland. All other chemicals and reagents were of analytical grade. Reverse osmosis (RO) water was used for all experiments.

### 2.2. Preparation of Navy Bean Materials

#### 2.2.1. Isolation of Free Starch Granules

Free starch granules were isolated from navy bean seeds according to the method described by Berg et al. [7].

#### 2.2.2. Isolation of Cotyledon Cells

Raw, intact cotyledon cells were isolated without gelatinising starch by successive treatments of navy beans with acid and then alkali according to the method described by Do et al. [10]. Dried navy bean seeds were soaked in a 0.1 M hydrochloric acid (HCl) solution (pH~1.3) at room temperature (~20 °C) for 24 h. The hydrated beans were manually dehulled to remove the outer seed coats and hypocotyls, split into cotyledons, and rinsed with RO water to remove the remaining acids. The cotyledons were subsequently soaked in a 0.06 M sodium hydroxide (NaOH) solution (pH~12.5) in 1 L Schott bottles. These bottles were placed in a shaking incubator and shaken at 20 °C and 150 rpm for 24 h. The softened, alkali-treated cotyledons were gently mashed by a pestle and mortar to a consistent paste. The resultant paste was successively passed through 150 and 53 μm certified test sieves (Endecotts Ltd., London, England) by extensive washing with water. The cell extract was collected on the 53 μm sieve and ready for pretreatment with pepsin.

#### 2.2.3. Enzymatic Removal of Intracellular Protein Matrix

Enzymatic removal of the intracellular protein matrix was carried out by means of prehydrolysis of isolated navy bean cotyledon cells (INCs) with pepsin. The cell extract (~20 g) collected on the 53 µm sieve (as described in Section 2.2.2) was mixed with pepsin solution (3.2 g pepsin in 400 mL of 0.034 M sodium chloride (NaCl) buffer, pH 2.0) in 1 L Schott bottles. These bottles were shaken in a shaking incubator at 37 °C and 100 rpm for 1, 4, or 24 h. The pH of the cell slurries was occasionally checked and, if necessary, adjusted to 2.0 with 1.0 M HCl.

After the prehydrolysisn step, the cell slurries were transferred to 50 mL centrifuge tubes and centrifuged at 1500× *g* for 10 min to recover the solids. The solid material was reslurried and washed with RO water before being recovered by centrifugation at 1500× *g* for 10 min. The washing and centrifugation procedure was repeated five times to remove the remaining acids and protein digests. The pepsin-treated cells were dehydrated by rinsing in three changes of absolute ethanol (1 g of extract per 5 mL of ethanol) for 5 min each at room temperature and recovered by centrifugation at 1800× *g* for 20 min. The cells were then spread onto clean glass plates and air-dried in a fume hood overnight at room temperature. The dried powder was bottled and stored at room temperature until further analysis.

INCs pretreated with pepsin for 1, 4, and 24 h were denoted as INC-1h, INC-4h, and INC-24h, respectively. A control sample (INC-Control) was prepared by treating the cell extract with pepsin-free NaCl buffer (0.034 M, pH 2.0) in the shaking incubator at 37 °C and 100 rpm for 24 h followed by ethanol dehydration and air drying. A native sample (INC-Native) was also prepared by subjecting the cell extract directly to the dehydration process without any chemical or enzymatic modification.

### 2.3. Determination of Physicochemical Properties

Moisture content was determined gravimetrically by drying the cell samples in an oven at 105 °C to a constant weight. Crude protein was analysed using the Dumas method (AOAC 968.06) [20] and a nitrogen-to-protein conversion factor of 6.25.

Total starch content was quantified using a total starch assay kit (K-TSTA). Total amylose content in starch was analysed using an amylose/amylopectin assay kit (K-AMYL). Both of the starch kits were obtained from Megazyme International Ireland Ltd., Wicklow, Ireland. The analysis was carried out according to the instructions given by the manufacturer. Chemical composition was expressed on a dry weight basis (dwb).

Swelling power and solubility of starch granules were determined after heating aqueous dispersions of INC, containing approximately 2% starch (*w*/*w*) at 90 °C for 30 min according to the method of Leach, McCowen, and Schoch [21].

### 2.4. Determination of α-Amylase Activity

Alpha-amylase activities of pancreatin (from porcine pancreas, 4× USP) and α-amylase (from porcine pancreas, type VI-B) were measured using the α-amylase enzymatic assay previously described by Bernfeld [22]. Enzyme solution was prepared by dissolving enzyme powder in RO water at a concentration of approximately 1 unit/mL of α-amylase. One-millilitre aliquots of the enzyme solution were added to 1 mL aliquots of soluble potato starch (1%, *w*/*v*) in 20 mM sodium phosphate buffer pH 6.9 in 15 mL Kimax screw-capped glass tubes. The capped tubes were mixed by swirling and placed in a water bath at 20 °C. After 3 min, the reaction was terminated by adding 1 mL of colour reagent solution (prepared by combining 8 mL of 5.3 M sodium potassium tartrate solution in 2 M NaOH with 20 mL of 96 mM 3,5-dinitrosalicylic acid and 12 mL of RO water) to each tube. The tubes were capped, mixed by swirling, and immediately incubated in a boiling water bath for 15 min. After cooling on ice for a few minutes, an additional 9 mL of RO water was added to each tube with inversion to mix the contents. The absorbance of the resulting coloured solution was measured spectrophotometrically at 540 nm. A blank assay was prepared by addition of 1 mL of the enzyme solution after adding the colour reagent and placing the tube in the boiling water bath. A standard calibration curve was prepared from a series of aqueous maltose solutions (0–2.0 mg/mL) and run parallel with the samples. The α-amylase activity was calculated and expressed in units of α-amylase per mg of enzyme powder. One unit (U) of amylase is defined as the amount of enzyme that liberates 1.0 mg of maltose from starch in 3 min at pH 6.9 and 20 °C.

### 2.5. In Vitro Starch Digestion

#### 2.5.1. Static In Vitro Starch Digestion Procedure

The static in vitro digestion protocol described by Dartois, Singh, Kaur, and Singh [23] was followed. This employed a two-stage method to simulate the gastric and small intestinal conditions for starch digestion. Simulated gastric and intestinal fluids (SGF and SIF) were prepared according to the US Pharmacopeia [24].

INCs were suspended in RO water in 400 mL glass beakers to obtain aqueous samples containing approximately 4% starch (*w*/*w*). The beakers were covered with aluminium foil and placed in a water bath at ~95 °C for 20 min to simulate cooking conditions. Cooked samples (~170 g) were subsequently cooled and introduced into 500 mL jacketed glass reactors. Temperature was maintained at 37 ± 1 °C by circulating water through the reactor jackets. pH was controlled using a pH meter and adjusted by manual addition of HCl (1.0 and 0.5 M) and/or NaOH (1.0 and 0.5 M). The content of each reactor was mechanically agitated using a magnetic stirrer bar at 300 rpm throughout digestion.

The reactor contents were first incubated with the SGF (17 mL) containing pepsin (enzyme/starch ratio, 1.765:100, *w*/*w*) at pH 1.2 for 30 min to simulate gastric digestion. After the gastric phase, the pH was adjusted to 6.8. The reactor contents were then incubated with the SIF containing pancreatin (enzyme/starch ratio, 1.3:100, *w*/*w*), amyloglucosidase (enzyme/starch ratio, 0.26:1, *v*/*w*), and invertase (enzyme/starch ratio, 1:1000, *w*/*w*) for 120 min to simulate small intestinal digestion.

Duplicate aliquots (0.5 mL) were withdrawn from the reactors after 1, 15, and 30 min of the gastric phase, and after 1, 5, 10, 15, 30, 60, 90, and 120 min of the small intestinal phase. The aliquots were immediately mixed with 2 mL of absolute ethanol in 15 mL centrifuge tubes to stop the enzymatic reaction. The resulting mixtures were vortex-mixed and centrifuged at 1800× *g* for 10 min. The ethanolic supernatants (0.1 mL) were incubated with 0.5 mL of amyloglucosidase and invertase in acetate buffer (0.1 mL amyloglucosidase and 3.75 mg invertase per 10 mL acetate buffer) at pH 5.2 and 37 °C for 10 min to completely convert soluble dextrins in the supernatants to glucose. The glucose released was quantified using a D-glucose assay kit (GOPOD-FORMAT, K-GLUC, Megazyme International Ireland Ltd., Wicklow, Ireland).

The percentage of starch hydrolysis at each sampling time point was used to construct hydrolysis curves and calculated using Equation (1):

(1) $$\%SH=\frac{S_{h}}{S_{i}}\times 100=0.9\times \frac{G}{S_{i}}$$

where %*SH* is the percentage of starch hydrolysis (%), $S_{h}$ is the amount of hydrolysed starch (g), $S_{i}$ is the initial amount of starch (g), and G is the amount of glucose released (g). A factor of 0.9 is used to convert glucose to starch and is based on the molecular mass ratio of starch monomer to glucose (162/180 = 0.9).

The initial rate of starch hydrolysis for the first 10 min of reaction (R10), according to Ezeogu, Duodu, and Taylor [25], was calculated using Equation (2):

(2) $$R10=\frac{m}{V\times t}$$

where m is the amount of starch hydrolysed (mg), V is the volume of reaction mixture at 10 min of the small intestinal digestion (mL), and t is the reaction time (t = 10 min).

#### 2.5.2. Effect of Proteolytic Enzymes on In Vitro Starch Digestion

**Figure 1:**
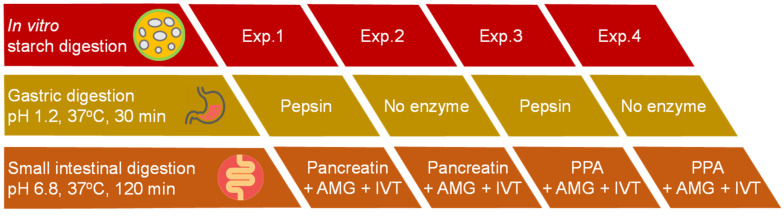
Schematic diagram of four different experiments for evaluating the indirect effect of proteolytic enzymes on in vitro starch digestion of cooked samples of control isolated navy bean cotyledon cells (INC-Control). Abbreviations: AMG—amyloglucosidase, IVT—invertase, PPA—porcine pancreatic α-amylase.

As illustrated in Figure 1, a set of four different experiments was designed to evaluate the indirect effect of proteolytic enzymes on in vitro digestion of starch in cooked samples of INC-Control. All experiments were performed in accordance with the in vitro digestion protocol suggested by Dartois et al. [23] (Section 2.5.1) but with different combinations of digestive proteases. All enzyme solutions were prepared freshly prior to analysis.

Four experiments (Exps.) were conducted as follows: 

Exp.1: control with gastric pepsin (GP) in the SGF followed by pancreatin containing pancreatin proteases (PP) in the SIF.

Exp.2: only PP in the SIF.

Exp.3: only GP in the SGF.

Exp.4: without any protease.

For Exps. 3 and 4, porcine pancreatic α-amylase (PPA) was used in place of pancreatin (a commercial mixture of amylase, lipase, and protease from porcine pancreas) to achieve the unit of α-amylase activity per mL of the final digestion mixture (U/mL) similar to that of pancreatin. The α-amylase activities (U/mg solid) of enzymes were determined using the protocol of Benfield [22] as detailed in Section 2.4. The pancreatin and PPA used in this study were found to exhibit an α-amylase activity of 35.9 ± 0.8 and 17.2 ± 0.6 U/mg solid, respectively.

### 2.6. Microscopy Analysis

For light microscopy (LM), INCs were mounted onto glass microscope slides, suspended in water, sealed with coverslips, and then viewed under an Axiophot light microscope (Carl Zeiss, Jena, Germany) operating in brightfield mode using the objective of 20× magnification. Representative light micrographs of cells were captured using a Leica DFC320 camera equipped with the Leica software application suite LAS V3.8 (Leica Microsystems, Wetzlar, Germany). Digesta samples taken after 0 and 120 min of the small intestinal digestion were stained with 2% (*w*/*v*) Lugol’s iodine solution and visualised under LM for detecting the presence of starch.

For scanning electron microscopy (SEM), INCs were directly mounted on double-sided adhesive tapes on aluminium stubs, sputter coated with gold (SCD 050, Balzers, Liechtenstein), and viewed under a scanning electron microscope (FEI Quanta 200 FEI Electron Optics, Eindhoven, the Netherlands). Representative electron micrographs of cell samples were captured with accelerating voltage of 25 kV and using the xT microscope software version 3.0.7 (FEI Quanta, Eindhoven, the Netherlands).

### 2.7. Statistical Analysis

Data were reported as means ± standard deviations for triplicate determinations unless otherwise specified. Tukey’s test and analysis of variance (ANOVA) were used to assess the significance of differences (*p* ≤ 0.05) between means using Minitab 18 software (Minitab Inc., State College, PA, USA).

## 3. Results and Discussion

### 3.1. Microstructural Characteristics of Isolated Navy Bean Cotyledon Cells

**Figure 2:**
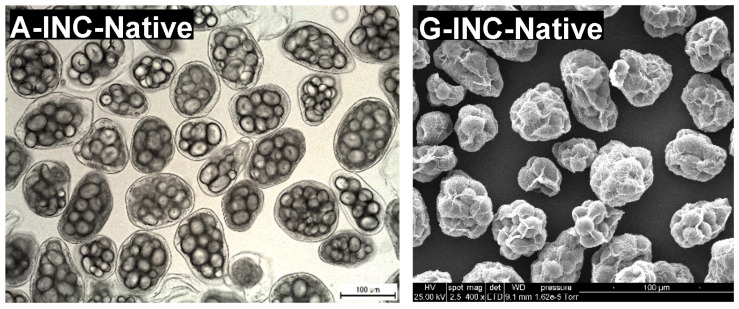

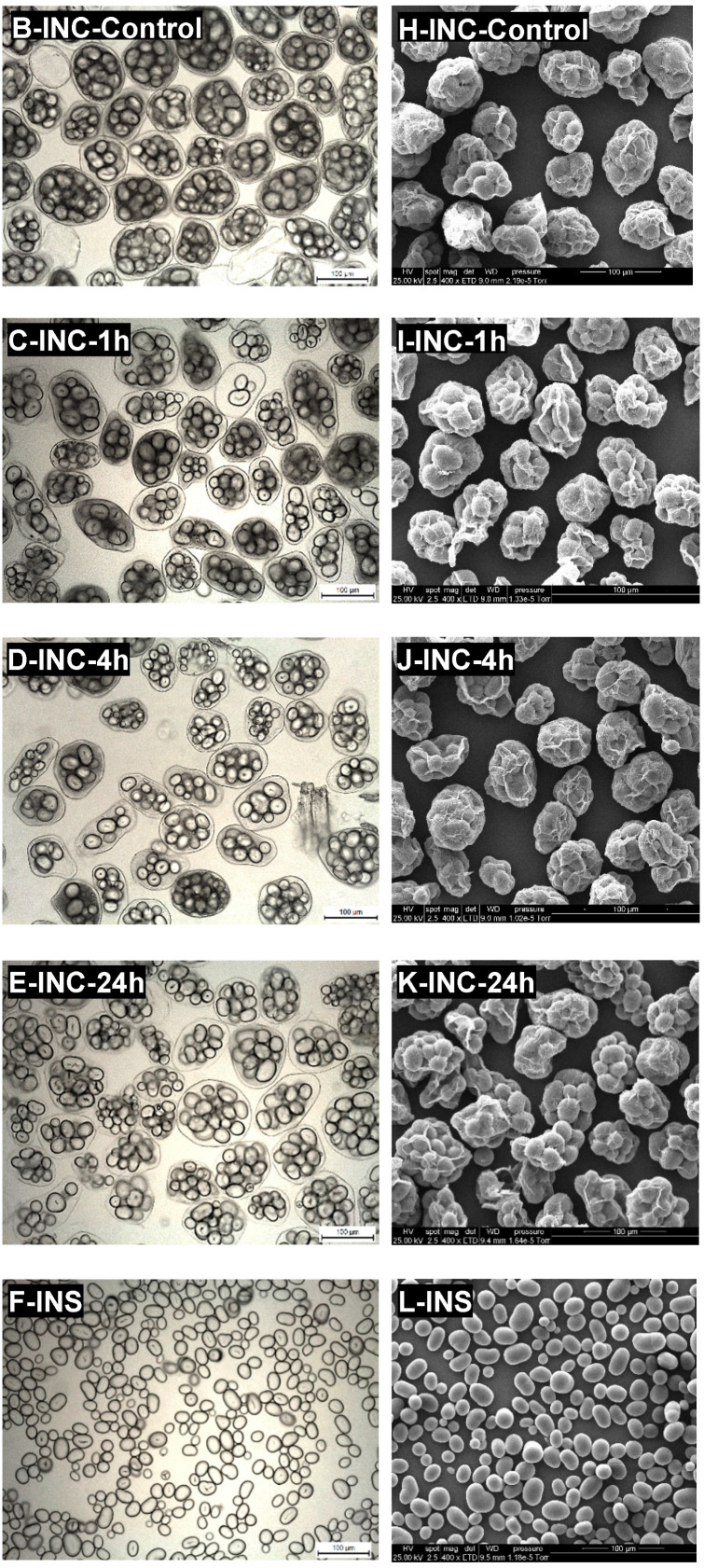
Representative brightfield light micrographs (**A**–**F**) and scanning electron micrographs (**G**–**L**) of isolated navy bean starch (INS), native INCs, control INCs, and INCs preincubated with pepsin for 1, 4, or 24 h at 37 °C and pH 2.0. Scale bar = 100 µm.

Isolated navy bean cotyledon cells (INC) were obtained using the sequential acid-alkali method (Do et al., 2019). This method enabled the easy separation of raw, intact cells without disrupting cell wall integrity and gelatinising starch. Representative light micrographs of INC (Figure 2A–E) confirmed good preservation of cell wall intactness and native starch granule structure after the cell isolation procedure and subsequent preincubation with pepsin. However, the cell samples contained some minor impurities, such as broken/damaged cells, free starch granules, and cell wall/protein fragments. Such impurities could have resulted from the breakage of the cell walls and consequent discharge of the cellular contents during the manual pestle-crushing process to separate the cells. In addition, the isolated navy bean starch (INS) sample was observed to be largely free of impurities (Figure 2F).

The microstructure of each native INC (Figure 2A) consists of multiple starch granules that are physically entrapped in a thick protein matrix and encased by an intact cell wall. The cotyledon cells and starch granules generally exhibit oval or round shapes. Similar microstructural observations of INCs have been reported previously [7,11]. No apparent morphological changes were detected in the control INCs (Figure 2B), indicating that the pepsin-free pretreatment of the native INCs did not significantly alter their cellular structures as expected.

Noticeably, the protein matrix appeared dark in contrast to the lighter background when viewed under LM (Figure 2A–D). The preincubation of the native INCs with pepsin at pH 2.0 and 37 °C resulted in the visual disappearance of the dark-coloured protein substance surrounding the starch granules, indicating hydrolysis of the protein matrix that had taken place inside the cells. As the preincubation time increased from 1 to 24 h, the protein matrix appeared progressively more transparent (lighter) (Figure 2C–E) and was no longer visually perceptible after 24 h (Figure 2E).

As is clearly shown by SEM micrographs in Figure 2, the INS (Figure 2L) displayed a smooth surface whereas the INCs (Figure 2G–K) possessed highly wrinkled surfaces, possibly due to extensive shrinkage and folding of the cell walls during the ethanol dehydration and air-drying process. The native starch granules were packaged into the INCs and wrapped around by the sheet-like cell walls while the protein matrix was not visually perceptible under SEM. These SEM observations agree well with those of previous reports [13,26]. Moreover, in line with the earlier LM observations, the cells seemed to have become progressively more transparent with longer exposure to pepsin, leading to greater visual clarity of the intracellular starch granules. It is possible that the protein matrix was partially removed from the cell cytoplasm when prehydrolysed with pepsin, leaving transparent cellular structures with distinct starch granules enclosed in loose cell walls.

### 3.2. Effect of the Intracellular Protein Matrix on Physicochemical Properties of Starch

The physicochemical properties of the INCs (control and pepsin-treated) and INS are presented in 1. The control INCs contained 64.3% starch and 17.6% protein (*w*/*w*, dwb). These values are within the range of previously published data [13]. Specifically, starch and protein are the two major components of legume cotyledon cells and constitute 57.2–69.0% and 17.3–20.3% of total dry cell mass, respectively [13].

Furthermore, it is evident from Table 1 that the preincubation with pepsin decreased the protein content while simultaneously increasing the total starch content of the INCs. As expected, a longer incubation time (1, 4, and 24 h) corresponded to a lower protein content (11.4, 9.2, and 2.7%) and a higher total starch content (70.5, 73.4, and 80.2%). The changes in the protein and starch contents were found to be statistically significant (*p* ≤ 0.05). These quantitative results support the earlier LM observations of the visual disappearance of the protein matrix in pepsin-treated cells. Additionally, INS contained 91.9% and 0.3% of starch and residual protein, respectively. The amylose content was similar (~28%) across all samples (*p* > 0.05), indicating that neither the pretreatment duration nor pepsin had any effect on the amylose content.

Moreover, Table 1 shows that the swelling power (SP) and starch solubility (SS) of INC-Control did not differ significantly from those of INC-1h or INC-4h (*p* > 0.05). Only prolonged incubation with pepsin up to 24 h could result in significantly higher levels of SP and SS (*p* ≤ 0.05). It has been suggested that the suppressed swelling and gelatinisation of starch granules entrapped in isolated cotyledon cells of legumes is attributed to limited water availability and spatial constraints inside cells [27,28]. This is perhaps a consequence of the physical confinement of starch granules in the cell wall/protein matrix as well as the competition for water and space with starch by nonstarch constituents such as proteins [13]. Therefore, as the prehydrolysis of INCs by pepsin progressed, more intracellular space that had been occupied by proteins was freed up. This might have allowed more water and free space to be made available inside cells for the swelling and dissolution of starch granules upon heating, resulting in greater leaching of soluble materials (mainly amylose) from the granules into the solution.

It is also worth noting that, a vast proportion of the protein matrix (~85%) was effectively removed from the cells by pepsin after 24 h. Despite this, INC-24h exhibited considerably lower SP and SS compared to INS from which the cell structural barriers had been completely removed. This implies that the remaining proteins and the intact cell walls of INC-24h prevented complete starch gelatinisation together. From these findings, it appears likely that the limited SP and SS of INCs are linked to the barrier effects exerted by the cell wall and the protein matrix. These physical barriers combine to inhibit the swelling and gelatinisation of the intracellular starch granules during hydrothermal processing.

### 3.3. Effect of the Intracellular Protein Matrix on In Vitro Starch Digestion

**Figure 3:**
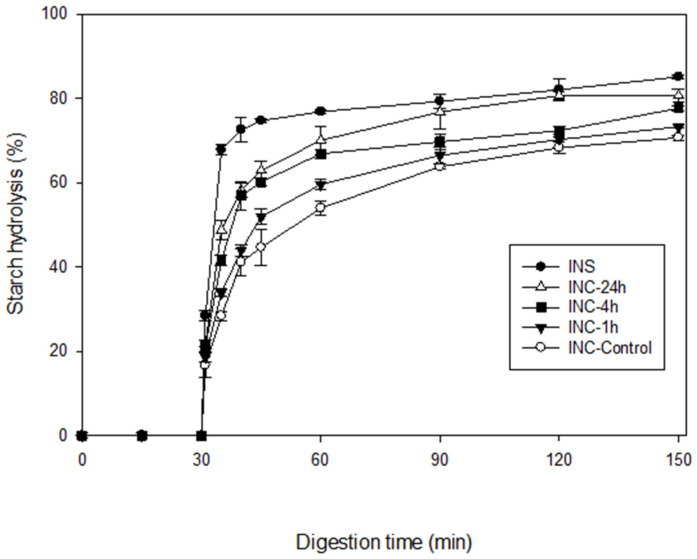
In vitro starch hydrolysis curves of cooked samples of control INCs (○) and INCs pretreated with pepsin for 1 h (▼), 4 h (■), and 24 h (△). INS (●) was included as a reference. Errors bars represent the standard deviations.

Cooked samples of the control and pepsin-treated INCs, differing in their protein content, were subjected to in vitro gastric and small intestinal digestion. A cooked INS sample was included as a reference. Starch hydrolysis curves are shown in Figure 3. As clearly seen in this figure, virtually no hydrolysis occurred during 30 min of gastric digestion due to the absence of starch-hydrolysing enzymes. Starch was hydrolysed by pancreatic α-amylase during 120 min of small intestinal digestion. During this phase, a general trend was observed in all samples with an increased percentage of hydrolysis during the early stages before reaching a plateau towards the end of digestion.

As noted in Table 2, statistically significant differences in the digestion kinetic parameters were found among the samples (*p* ≤ 0.05). The percentage of starch hydrolysed at 120 min of small intestinal digestion (H120, %) decreased in the following order: INS (85.1) > INC-24h (80.6) > INC-4h (77.6) > INC-1h (73.2) > INC-Control (70.6). In a somewhat similar pattern to that seen in H120, the initial rate of starch hydrolysis (R10, mg/mL/min) calculated for the first 10 min of reaction when it was highest decreased in the following order: INS (2.76) > INC-24h (2.11) ≈ INC-4h (2.07) > INC-1h (1.50) ≈ INC-Control (1.40). Evidently, both R10 and H120 progressively increased as the duration of the pepsin pretreatment increased from 1 to 24 h. These results demonstrate that the enzymatic removal of the protein matrix improves the rate of amylolysis at the initial stages of in vitro digestion and ultimately enhances the starch digestibility of INCs. This clearly proves that, aside from the intact cell wall, the protein matrix acts as an additional barrier to α-amylolysis of the enclosed starch. Similar findings have been previously reported for kidney bean cotyledon cells by Rovalino-Córdova et al. [19], although the conditions under which the cells were isolated by heating and then pretreated with proteases were different to those used in the present study.

**Figure 4:**
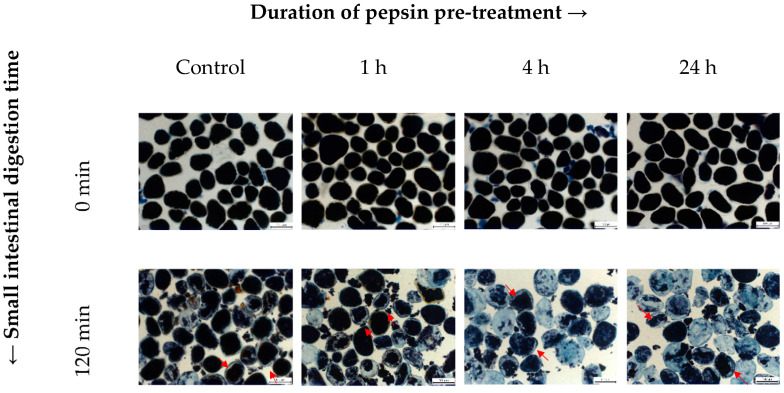
Representative brightfield light micrographs taken at 0 and 120 min of small intestinal digestion of control INCs and INCs pretreated with pepsin for 1, 4, and 24 h. The cells were stained with Lugol’s iodine reagent for detecting the presence of starch. Red arrows indicate empty “gap spaces”. Scale bar = 100 µm.

Figure 4 shows representative light micrographs of INC samples before and after 120 min of the simulated small intestinal digestion. As can be seen from this figure, most cells maintained their structural integrity throughout in vitro digestion whereas only a minor portion of cells had broken and released their contents. In addition, a general trend was observed in all samples. Specifically, most cells became “emptier” after 120 min with the formation of empty “gap spaces” between the cellular contents and the peripheral cell walls. A possible explanation for this, which we have previously proposed [11], is that the starch hydrolysis by α-amylase progresses from the periphery towards the centre of the cells and, thus, causes “shrinking”/emptying of the cellular contents.

Staining of INCs with iodine enabled the visualisation of changes in the starch content. The blue–black colour intensity of amylose-iodine complexes is thought to be proportional to the amount of starch present in the cells, thus providing qualitative information on the extent of starch hydrolysis at specific digestion times. Emptying of the cellular contents and a decrease in iodine staining intensity were observed after 120 min in all samples. This effect occurred to a much greater extent in those with a longer duration of pepsin pretreatment. Evidently, in comparison with INCs preincubated with pepsin for only 1 h, those preincubated for 4 h or longer exhibited emptier cellular contents and greater reductions of iodine staining intensity after their exposure to small intestinal conditions, supporting their higher extents of amylolysis (H120) determined quantitatively. These findings are in line with previous reports investigating in vitro starch digestion kinetics of legume cotyledon cells [17,18,29].

It is also noteworthy that the pretreatment of native INC with pepsin resulted in only partial protein hydrolysis. After 24 h, approximately 85% of protein digestion was achieved as calculated from Table 2. Despite the effective enzymatic removal of the bulk of the protein matrix, INC-24h had significantly lower values for R10 and H120 than INS (deprived entirely of cell structural barriers). This is mostly due to the retention of the intact cell walls in the INC-24h, providing a diffusion barrier to the passage of α-amylase. This evidence implies that the barrier effects of the protein matrix and the cell wall combine to inhibit in vitro starch digestion.

In an attempt to separate the net contribution towards the barrier effect of the protein matrix from that of the intact cell wall, Rovalino-Córdova et al. [19] incubated kidney bean cotyledon cells with proteases for 20 h in order to completely hydrolyse proteins prior to in vitro starch digestion with α-amylase. Contrary to expectations, it was noted that only 50% protein hydrolysis was achieved despite the prolonged protease pretreatment. Nevertheless, protease-treated cells showed a higher rate of starch hydrolysis at early digestion times compared to control cells (without protease pretreatment). These results are consistent with our findings showing the protein matrix as an additional barrier to in vitro starch digestion, but its individual contribution could not be quantitatively determined due to the incomplete protein prehydrolysis.

The starch–protein matrix, consisting of a compact protein matrix entrapping starch granules, is a distinctive microstructural feature of some natural and processed foods. Starch–protein interactions and physiochemical characteristics of the protein matrix (e.g., gluten network in pasta, disulphide-bonded kafirin network in sorghum, etc.) may play a pivotal role in retarding α-amylase digestion of starch, resulting in foods with slow starch digestion properties and potential to modulate glycaemic response [30,31,32]. However, due to the low content of sulphur amino acids in bean proteins, the slow starch digestion of INCs may be attributed primarily to the microstructural organisation of starch granules and proteins in the cell cytoplasm rather than the formation of disulphide bonds in proteins [19]. In fact, INCs can be represented as a double-layered encapsulation system. The protein matrix acts as an inner layer for coating starch granules while the cell wall acts as an outer layer for coating the starch granule–protein matrix. These dual encapsulation layers provide double protection against amylolytic degradation, perhaps through limiting the swelling/gelatinisation of starch granules (as evidenced by SP and SS data presented in Table 1, Section 3.2) as well as hindering α-amylase access/binding to starch. Therefore, the breakdown of either the cell wall or protein barriers can lead to a substantial increase in the rate and extent of starch hydrolysis as has been demonstrated in this and other studies [7,17,18,19,33].

### 3.4. Effect of Proteolytic Enzymes on In Vitro Starch Digestion

As demonstrated above, the protein matrix plays a crucial part in limiting in vitro digestion of starch in INCs. Since dietary proteins are susceptible to proteolysis catalysed by GP in the SGF and PP (mainly trypsin and chymotrypsin) in the SIF, it is necessary to further explore the indirect effect of these proteolytic enzymes on starch digestion.

**Figure 5:**
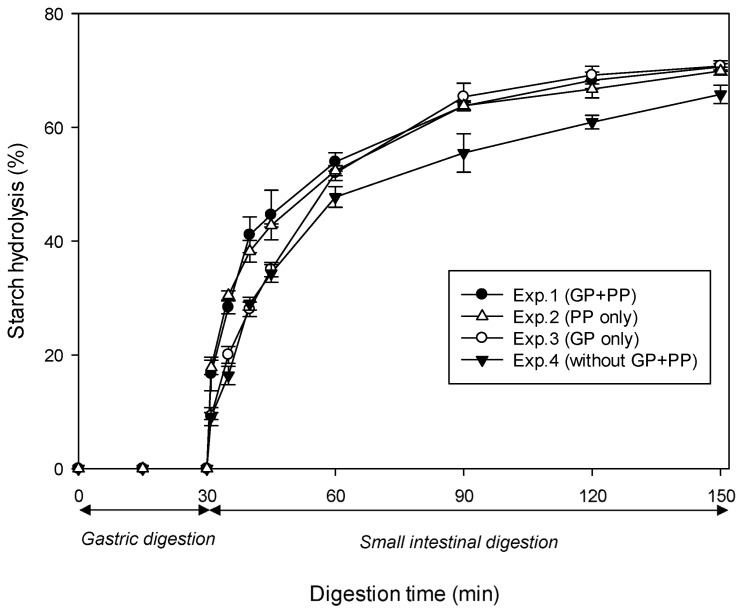
In vitro starch hydrolysis curves of cooked samples of control INCs from four experiments differing in protease combinations. Symbols (●, △, ○, ▼) represent the four experiments (see Experimental Section 2.5.2). Errors bars represent the standard deviations. Abbreviations: GP—gastric pepsin, PP—pancreatin proteases.

Figure 5 shows in vitro starch digestion curves of cooked samples of INC-Control from four experiments differing in the combination of proteases in the digestive fluids: (Exp.1) GP followed by PP, (Exp.2) GP alone, (Exp.3) PP alone, and (Exp.4) no protease. As evident in this figure, all four combinations displayed a similar starch hydrolysis pattern. Specifically, virtually no starch was broken down during 30 min of gastric digestion. This was followed by a gradual rise in the percentage of hydrolysis during 120 min of small intestinal digestion. As shown in Table 3, the initial rate of amylolysis (R10, mg/mL/min) decreased in the following order: Exp.1 (1.40) ≈ Exp.3 (1.41) > Exp.2 (0.97) ≈ Exp.4 (1.04). Meanwhile, the extent of amylolysis (H120, %) decreased in the following order: Exp.1 (70.6) ≈ Exp.2 (70.8) ≈ Exp.3 (69.9) > Exp.4 (65.8).

The quantitative data clearly show that both R10 and H120 values were significantly reduced when cooked control INCs were digested in the absence of all proteases. This is consistent with previous work conducted by Rovalino-Córdova et al. [18,19]. These authors found a significant decrease in the rate and extent of α-amylase digestion of starch when kidney bean cotyledon cells were digested with the exclusion of all proteolytic enzymes (i.e., pepsin in the SGF, trypsin and chymotrypsin in the SIF). Wang, Li, Zhang, Wang, and Copeland [34] also found that the greatest starch digestibility of cooked rice was achieved through a combination of amylolytic enzymes (PPA and AMG) and proteolytic enzymes (pepsin, trypsin, and chymotrypsin). Conversely, the total exclusion of all proteases resulted in the lowest starch digestibility. These findings have demonstrated that efficient enzymatic hydrolysis of the protein matrix is necessary for improving starch digestibility, which has previously been described as a cooperative process [18]. In addition, digestion with only PP resulted in a higher R10 value but a similar H120 value compared to digestion with only GP, which may be suggestive of the effectiveness of different proteolytic enzymes in degrading the protein matrix. This is supported by the previous suggestion that PP are thought to be more efficient in hydrolysing dietary proteins than GP [19].

Overall, the results of this study have provided strong evidence that the natural presence of proteolytic enzymes in both the stomach and the small intestine of the human GI tract is necessary for efficient in vitro digestion of starch inside INCs. Initially, GP is likely to play a role in loosening the compact starch granule–protein matrix. Subsequently, extensive protein degradation by PP in the small intestine may facilitate enzyme mobility within the cell cytoplasm and enhance starch–amylase interactions. Considering the facilitating effect of proteolytic enzymes on starch digestion, the encapsulation of starch granules in double-layered INC with the protein matrix (inner layer) being surrounded by the cell wall (outer layer) seems to be advantageous in the sense that the protein matrix is shielded from proteases, making it more resistant to proteolysis while providing extra protection for starch.

## 4. Conclusions

The present study has unveiled intriguing new insights into the role of the protein matrix in modulating the in vitro digestion of starch in navy bean cotyledon cells. We have shown that the entrapment of starch granules in the protein matrix may restrict their swelling and gelatinisation. In addition to the cell wall, the protein matrix presents a secondary physical barrier blocking the access/binding of α-amylase to starch. Proteolytic degradation of the protein matrix removes these restrictions, rendering starch more susceptible to α-amylolysis. Hence, efficient protein hydrolysis before or during simulated GI digestion is a necessary prerequisite for improving starch digestibility. Finally, as proposed by Do, Singh, Oey, and Singh [35], the unique cotyledon cell structure serves as a great source of inspiration for designing biomimetic materials. Encapsulation of starch granules within a protein core and a polysaccharide shell to form a double-layered structure is a novel strategy to fabricate novel food-grade particles for reduced glycaemic impact.

## Figures and Tables

**Table 1:** Physicochemical properties of INS, control INCs, and INCs pretreated with pepsin for 1, 4, or 24 h.

Navy Bean Material	AM (%)	TS (% dwb)	P (% dwb)	SP (g/g)	SS (%)
INC-Control	28.8 ± 0.0 ^a^	64.3 ± 0.5 ^e^	17.6 ± 0.7 ^a^	5.2 ± 0.1 ^d^	5.5 ± 0.0 ^c^
INC-1h	28.6 ± 0.2 ^a^	70.5 ± 0.4 ^d^	11.4 ± 0.3 ^b^	5.4 ± 0.1 ^cd^	5.5 ± 0.0 ^c^
INC-4h	27.8 ± 0.3 ^a^	73.4 ± 0.2 ^c^	9.2 ± 0.3 ^c^	5.6 ± 0.0 ^c^	6.1 ± 0.1 ^c^
INC-24h	29.0 ± 0.0 ^a^	80.2 ± 0.5 ^b^	2.7 ± 0.2 ^d^	7.4 ± 0.2 ^b^	8.3 ± 0.3 ^b^
INS	28.0 ± 0.7 ^a^	91.9 ± 0.9 ^a^	0.3 ± 0.2 ^e^	12.6 ± 0.1 ^a^	16.9 ± 0.2 ^a^

^a,b,c,d,e^ Values are means ± standard deviations of three determinations. Values with the same subscripts in a column do not differ significantly (*p* > 0.05). Abbreviations: AM—amylose content, TS—total starch content, P—protein content, SP—swelling power, SS—starch solubility.

**Table 2:** Kinetic parameters of in vitro starch digestion of cooked samples of INS, control INCs, and pepsin-treated INCs.

Navy Bean Materials	R10 (mg/mL/min) ^f^	H120 (%) ^g^
INC-Control	1.40 ± 0.11 ^c^	70.6 ± 0.6 ^e^
INC-1h	1.50 ± 0.03 ^c^	73.2 ± 0.3 ^d^
INC-4h	2.07 ± 0.13 ^b^	77.6 ± 0.6 ^c^
INC-24h	2.11 ± 0.13 ^b^	80.6 ± 1.6 ^b^
INS	2.76 ± 0.13 ^a^	85.1 ± 0.4 ^a^

^a,b,c,d,e^ Values are means ± standard deviations of three determinations. Values with the same subscripts in a column do not differ significantly (*p* > 0.05). ^f^ R10: initial rate of starch hydrolysis calculated for the first 10 min of reaction. ^g^ H120: values for percentage of starch hydrolysis determined experimentally at 120 min of small intestinal digestion.

**Table 3:** Kinetic parameters of in vitro starch digestion of cooked samples of control INCs from four experiments differing in protease combinations.

Experiments	R10 (mg/mL/min) ^c^	H120 (%) ^d^
Exp.1 (GP + PP)	1.40 ± 0.11 ^a^	70.6 ± 0.6 ^a^
Exp.2 (PP only)	1.41 ± 0.07 ^a^	69.9 ± 0.7 ^a^
Exp.3 (GP only)	0.97 ± 0.04 ^b^	70.8 ± 0.9 ^a^
Exp.4 (without GP + PP)	1.04 ± 0.04 ^b^	65.8 ± 1.6 ^b^

^a,b^ Values are means ± standard deviations of three determinations. Values with the same subscripts in a column do not differ significantly (*p* > 0.05). ^c^ R10: initial rate of starch hydrolysis calculated for the first 10 min of reaction. ^d^ H120: values for percentage of starch hydrolysis determined experimentally at 120 min of small intestinal digestion.

## Data Availability

Not applicable.
